# HYAL-2–WWOX–SMAD4 Signaling in Cell Death and Anticancer Response

**DOI:** 10.3389/fcell.2016.00141

**Published:** 2016-12-06

**Authors:** Li-Jin Hsu, Ming-Fu Chiang, Chun-I Sze, Wan-Pei Su, Ye Vone Yap, I-Ting Lee, Hsiang-Ling Kuo, Nan-Shan Chang

**Affiliations:** ^1^Department of Medical Laboratory Science and Biotechnology, National Cheng Kung UniversityTainan, Taiwan; ^2^Department of Neurosurgery, Mackay Memorial Hospital, Mackay Medicine, Nursing and Management College, and Graduate Institute of Injury Prevention and Control, Taipei Medical UniversityTaipei, Taiwan; ^3^Department of Cell Biology and Anatomy, College of Medicine, National Cheng Kung UniversityTainan, Taiwan; ^4^Institute of Molecular Medicine, College of Medicine, National Cheng Kung UniversityTainan, Taiwan; ^5^Advanced Optoelectronic Technology Center, National Cheng Kung UniversityTainan, Taiwan; ^6^Center of Infectious Disease and Signaling Research, College of Medicine, National Cheng Kung UniversityTainan, Taiwan; ^7^Department of Neurochemistry, New York State Institute for Basic Research in Developmental DisabilitiesStaten Island, NY, USA; ^8^Graduate Institute of Biomedical Sciences, College of Medicine, China Medical UniversityTaichung, Taiwan

**Keywords:** Zfra, cancer, hyaluronan, hyaluronidase, prevention, treatment, HYAL-2, Z cell

## Abstract

Hyaluronidase HYAL-2 is a membrane-anchored protein and also localizes, in part, in the lysosome. Recent study from animal models revealed that both HYAL-1 and HYAL-2 are essential for the metabolism of hyaluronan (HA). *Hyal-2* deficiency is associated with chronic thrombotic microangiopathy with hemolytic anemia in mice due to over accumulation of high molecular size HA. HYAL-2 is essential for platelet generation. Membrane HYAL-2 degrades HA bound by co-receptor CD44. Also, in a non-canonical signal pathway, HYAL-2 serves as a receptor for transforming growth factor beta (TGF-β) to signal with downstream tumor suppressors WWOX and SMAD4 to control gene transcription. When SMAD4 responsive element is overly driven by the HYAL-2–WWOX–SMAD4 signaling complex, cell death occurs. When rats are subjected to traumatic brain injury, over accumulation of a HYAL-2–WWOX complex occurs in the nucleus to cause neuronal death. HA induces the signaling of HYAL-2–WWOX–SMAD4 and relocation of the signaling complex to the nucleus. If the signaling complex is overexpressed, bubbling cell death occurs in WWOX-expressing cells. In addition, a small synthetic peptide Zfra (zinc finger-like protein that regulates apoptosis) binds membrane HYAL-2 of non-T/non-B spleen HYAL-2^+^ CD3^−^ CD19^−^ Z lymphocytes and activates the cells to generate memory anticancer response against many types of cancer cells *in vivo*. Whether the HYAL-2–WWOX–SMAD4 signaling complex is involved is discussed. In this review and opinion article, we have updated the current knowledge of HA, HYAL-2 and WWOX, HYAL-2–WWOX–SMAD4 signaling, bubbling cell death, and Z cell activation for memory anticancer response.

## Introduction

High molecular weight hyaluronan (HA) is accounted for approximately 0.02% of a human body weight (e.g., 16 g HA in an 80 kg individual), in which one third of the amount undergoes daily turnover (Stern, 2004; Viola et al., 2015; Chanmee et al., 2016). Hyaluronidases play a key role in the catabolism of HA. Apparently, the highly active turnover of HA is of physiological significance, and degraded small HA fragments play important physiological and pathological roles such as in cancer metastasis (Bertrand et al., 1992; Josefsson et al., 2011; Lokeshwar et al., 2014). Urinary excretion of degraded HA of 400–12,000 Daltons is around 330 μg daily (Laurent et al., 1987). Excessive amounts of small HA fragments can be found in urine, plasma and cancer lesions from cancer patients (Laurent et al., 1987; Turley et al., 2016). HA is accumulated in the extracellular matrix in the brains of patients suffering neurodegeneration (Sherman et al., 2015). The elevated HA accumulation in the brain may occur as a result of injury to neurons, glial cells and surrounding brain tissues (Sherman et al., 2015). HA contributes to the control of cell proliferation, migration and differentiation (Sherman et al., 2015; Turley et al., 2016). A recent study showed that HA does not contribute to the growth and differentiation of human epidermal keratinocyte (Malaisse et al., 2016). Nevertheless, UDP-glucose induces HA synthesis in keratinocytes via P2Y14 receptor, STAT3 signaling and activation of hyaluronan synthase (HAS) promoter (Jokela et al., 2014). The conformation of HA in solution is readily subjected to changes upon altering the compositions of solvents, and this affects the functional properties of HA in many biological processes such as interactions with complement proteins and their associated hemolytic function (Chang and Boackle, 1985; Chang et al., 1985; Hong et al., 2007b). For example, under low ionic strength conditions, HA has a less compact conformation in the saliva that allows its interactions with complement proteins and bacteria (Chang and Boackle, 1986).

## HA and hyaluronan synthase (HAS)

HA is a non-sulfated glycosaminoglycan, consisting of repeating disaccharide units of D-glucuronic acid and D-N-acetylglucosamine, which links through alternating beta-1,4 and beta-1,3 glycosidic bonds (Laurent et al., 1987; Cyphert et al., 2015; Turley et al., 2016). The disaccharide unit of HA repeats 10,000 or more times, reaching a molecular mass of ~4 million Daltons (each disaccharide ~ 400 Daltons; Yeh et al., 2011). HA chains are highly hydrophilic and have hydrophobic patches. HA forms twofold helix, expands tremendously in aqueous solutions, and forms a meshwork-liked tertiary structure through β-sheets (Heatley and Scott, 1988; Scott et al., 1991). High-molecular-mass HA (10^5^ saccharides, 2 × 10^4^ kDa) in synovial fluid and vitreous humor may act like a barrier to other molecules and cells, so they have anti-angiogenic, anti-inflammatory, and immunosuppressive effects. Indeed, fairly high molecular mass of HA, which is more than 5 times larger than human or mouse HA, confers resistance to cancer in naked mole rat (Tian et al., 2013). Small HA fragments induce the release of inflammatory chemokines, stimulate CD44 cleavage, increase angiogenesis, and promote tumor cell migration (Ghosh et al., 2015; Monslow et al., 2015).

HA distribution and abundance in tissues is tightly regulated by hyaluronan synthases (HAS1, HAS2, and HAS3) and hyaluronidases (HYAL1, HYAL2, and HYAL3). HAS are membrane-bound glycosyltransferases that act on alternating UDP-α-D-glucuronate (from pentose phosphate pathway) and UDP-α-N-acetyl-D-glucosamine (from glycolysis) to produce HA (Lee and Spicer, 2000; Itano and Kimata, 2002). HAS1, for example, adds new intracellular sugar-UDPs at the reducing end of growing hyaluronyl-UDP chains (Weigel, 2015). Presence of heterodimeric complexes of HAS1-HAS2, HAS2-HAS2, and HAS2-HAS3 has been reported (Bart et al., 2015). As established in *Has* knockout mice, Has2 is important for embryo development, while Has1 and Has3 have no effects (Camenisch et al., 2000; Bai et al., 2005). Has1 is frequently upregulated in inflammatory diseases such as atherosclerosis, osteoarthritis, and infectious lung diseases (Siiskonen et al., 2015). By using recombinant HAS, synthesized HA is 2 × 10^5^–2 × 10^6^ Daltons by HAS1, 2 × 10^6^ by HAS2, and 10^5^–10^6^ by HAS3 (Itano and Kimata, 2002). Deficiency of HAS and hyaluronidases contributes to numerous types of diseases. For example, mucopolysaccharidosis IX is due to lack of HYAL-1 and HAS2 in a single person having cardiac pathology (Triggs-Raine and Natowicz, 2015).

It is generally believed that high molecular weight HA provides a space-filling function for tissues and organs (Lee and Spicer, 2000). In this case, HA is strong in anti-inflammation, anti-angiogenesis and anti-cancer growth, and supports wound healing (Tian et al., 2013; Tolg et al., 2014; Schwertfeger et al., 2015; Litwiniuk et al., 2016). In contrast, low molecular weight HA is capable of stimulating angiogenesis, provoking proinflammation, and supporting cancer growth (Tian et al., 2013; Schwertfeger et al., 2015; Litwiniuk et al., 2016). These aforementioned scenarios may not necessarily be true. The long-chain HA can be physically altered and partially degraded. Due to the altered conformation and reduced sizes, HA is able to achieve a great potency in anti-inflammation and blocking cancer growth (Chang and Su, 2016).

## HA receptors and signaling

There are many HA receptors identified, whereas their binding affinities with native HA, conformationally altered HA, and HA fragments have been poorly defined. Among the most studied receptor proteins are CD44 and receptor for hyaluronan-mediated motility (RHAMM), as they participate in inflammation and cancer motility, migration and metastasis (Slevin et al., 2007; Lokeshwar et al., 2014; Tolg et al., 2014; Misra et al., 2015). Furthermore, both CD44 and RHAMM are associated with the development of stem cell and cancer stem cell (Jiang et al., 2013; Shigeishi et al., 2013; Kouvidi et al., 2014; Jordan et al., 2015). FAK/SRC-mediated ERK activation is involved in signaling event for stem cell development. Additional receptor proteins for HA include intracellular adhesion molecule-1 (ICAM-1) (Bruynzeel et al., 1993), hyaluronan receptor for endocytosis (HARE) (Pandey and Weigel, 2014), and lymphatic vessel endothelial hyaluronan receptor (LYVE)-1 (Banerji et al., 1999; Lawrance et al., 2016). Clustering of LYVE-1 is essential for HA binding in the lymphatic endothelial cells (Lawrance et al., 2016). The sizes of HA affect CD44 clustering (Yang et al., 2012). Notably, CD147 (also known as emmprin or basigin) is shown to control HA synthesis and interact with the HA receptors CD44 and LYVE-1. These interactions appear to contribute to drug transporter-associated chemoresistance (Grass et al., 2014).

## Hyaluronidases and clinical relevance and applications

Hyaluronidases cleave the β-1,4-glucosaminidic bond between glucosamine and glucuronic acid (Girish and Kemparaju, 2007). These enzymes have been widely utilized in clinical applications. Hyaluronidases are utilized as adjuvants to destruct the extracellular matrix to enhance the penetration of drugs to target areas in the body (Buhren et al., 2016). Six hyaluronidase-like genes in humans have been identified, which are *HYAL1, HYAL2, HYAL3, HYAL4, PH-20/SPAM1*, and a pseudogene *HYALP1* (Csoka et al., 2001; Stern and Jedrzejas, 2006). *HYAL1, HYAL2*, and *HYAL3* are clustered on the chromosome 3p21.3, and *HYAL4, HYALP1* (a pseudogene), and *SPAM1* (sperm adhesion molecule 1) on chromosome 7p31.3. HYAL-1 is present in many tissues and found in the circulation, and is internalized by monocytes and endothelial cells to relocate in the lysosomes (Frost et al., 1997; Csoka et al., 2001; Puissant et al., 2014). HYAL-1 is functionally active at low pH (pH = 3.8). Hyaluronidases HYAL-1 and PH-20, but not HYAL-2, can be found in secretion.

HYAL-2 has a 20-amino-acid signal sequence at the *C*-terminus that allows the formation of a GPI linkage onto the cell membrane (Rai et al., 2001). Rai et al. showed that HYAL-2 acts as a receptor for jaagsiekte sheep retrovirus. Functionally, membrane HYAL-2 degrades HA bound by co-receptor CD44 to generate end products of approximately 20 kDa fragments. That is, CD44 assists HYAL-2 in degrading HA. The HA fragments can be endocytosed, and then HYAL-1 further degrades the HA fragments (Puissant et al., 2014).

HYAL-1, but not HYAL-3, plays an important role in controlling ovarian folliculogenesis by acting on the follistatin/activin/Smad3 pathway (Dumaresq-Doiron et al., 2012). No *HYAL2* deficiency has been found in human. *Hyal2*-deficient mice exhibit accumulation of extremely large HA molecules in tissues and in circulation, and this contributes to the development of chronic thrombotic microangiopathy (Onclinx et al., 2015) and mild craniofacial and vertebral abnormalities (Bourguignon and Flamion, 2016). Megakaryocyte-derived HYAL-2 causes HA degradation, which is needed for thrombopoiesis (Petrey et al., 2016). HYAL-3 lacks an intrinsic hyaluronidase activity in somatic cells *in vivo*, whereas it participates in the hyaluronan metabolism by augmenting the activity of HYAL-1 (Hemming et al., 2008). HYAL-3 is abundant in the testis, and the enzymatic activity has been detected in spermatozoa (Reese et al., 2010). PH-20, also known as SPAM1, is a GPI-anchored hyaluronidase, which is expressed in the lysosome-derived acrosome on a sperm's surface. Recombinant human PH-20 has been used clinically to enhance drug penetration to cancer target (Frost, 2007). PH-20 facilitates fertilization by enhancing penetration of the sperm to the ovum via cleavage of the extracellular matrix (Girish and Kemparaju, 2007). PH-20 is expressed in sperms and breast cells (Beech et al., 2002), and is regarded as a tumor marker for laryngeal cancers (Godin et al., 2000).

## HA degradation without catalytic enzymes

HA can be degraded under enzyme-free conditions, including acid hydrolysis (Hair et al., 2010), heat treatment by autoclave (Pichi et al., 2012), sonication (Kubo et al., 1993), and free-radical-based cleavage (Hrabárová et al., 2012). By sonication using a fixed intensity at various durations, HA is broken down to a constant size at 11 kDa after sonication. The main sugar residues at the reducing and non-reducing termini of depolymerized HA are N-acetylglucosamine (86%) and glucuronic acid (98%), respectively (Kubo et al., 1993). In contrast, we have shown that sonicated, degraded HA exhibits as a broad range of molecular sizes from 10 to 200 kDa (Chang and Su, 2016). The likely difference from the above report is due to using a different type of sonication machine and the energy of sonication used.

## Role of HA and hyaluronidases in cancer progression and metastasis

Overproduction of HA and hyaluronidases is frequently seen in cancer cells. This correlates with increased cancer malignancy and metastatic potential (Sironen et al., 2011; Shepard, 2015; Sato et al., 2016). Depletion of HA is a doable approach to suppress cancer growth (Simpson et al., 2002). The amount of HA surrounding invasive tumors is higher than that in non-invasive tumors (Bertrand et al., 1992), suggesting that HA is one of the reliable markers for tumor progression and malignancy (Gritsenko et al., 2012). It has been shown that excessive levels of HAS enzymes induce accumulation of HA in breast cancer, and that these enzymes are associated with tumor aggressiveness and poor patient outcome (Auvinen et al., 2014). Conceivably, peritumor HA is subjected to digestion by membrane HYAL-2 with the assistance of CD44. The resulting small HA molecules undergo conformational changes by de-coupling and re-coupling of long and short chains due to local thermal changes by cancer-associated inflammation. The resulting molecular hybrids are the likely stimulators for tumor growth, and they may act as auto-chemotactic factors for increasing cancer cell mobility (Saito et al., 2011).

## Tumor suppressor WWOX is anchored on the cell membrane by HYAL-2

Hyaluronidases PH-20, HYAL-1, and HYAL-2 induce the expression of tumor suppressor WWOX in fibroblasts (Chang et al., 2001, 2007; Chang, 2002). WW domain-containing oxidoreductase, known as WWOX, FOR, and WOX1, was first isolated in 2000 independently by 3 groups (Bednarek et al., 2000; Ried et al., 2000; Chang et al., 2001). Human *WWOX* gene has 1.1 million bases and is located on a chromosomal common fragile site 16q23 or *FRA16D*. High frequency of loss of heterozygosity (LOH) of *WWOX* gene at approximately 30–50% levels has been shown in many types of cancer cells (Aqeilan and Croce, 2007; Chang et al., 2007; Del Mare et al., 2009; Gardenswartz and Aqeilan, 2014; Abu-Remaileh et al., 2015; Chang, 2015; Chang et al., 2015; Richards et al., 2015; Schrock and Huebner, 2015). Mutation of *WWOX* gene in breast cancer occurs frequently at exons 4–9 (Ekizoglu et al., 2012). Loss of *WWOX* gene expression can be due to hypermethylation of gene promoter (Iliopoulos et al., 2005; Chang et al., 2010; Gardenswartz and Aqeilan, 2014). When cytosolic WWOX protein relocates to the nucleus under stress conditions, WWOX is able to maintain genomic stability by controlling ATM activation and DNA damage response (Abu-Odeh et al., 2014; Abu-Remaileh et al., 2015). Most recently, WWOX-mediated suppression of cancer cell growth has been established in *Drosophila* (O'Keefe et al., 2015). Notably, null mutations of *WWOX/Wwox* gene in humans and animals lead to severe neural diseases (e.g., microcephaly, seizure, ataxia, etc.), growth retardation, metabolic disorders, developmental delay, and early death (Aldaz et al., 2014; Chang et al., 2014; Alkhateeb et al., 2016; Elsaadany et al., 2016). Nevertheless, no spontaneous cancer formation has been seen in the newborns, arguing whether WWOX is a real tumor suppressor.

WWOX possesses two *N*-terminal WW domains (containing conserved tryptophan residues), a nuclear localization sequence, and a *C*-terminal short-chain alcohol dehydrogenase/reductase (ADH/SDR) domain (Bednarek et al., 2000; Ried et al., 2000; Chang et al., 2001). Activation of WWOX via Tyr33 phosphorylation is essential for its proapoptotic function and anticancer property. Under apoptotic stress, WWOX undergoes Tyr33 phosphorylation, binds pS46-p53, and translocates with pS46-p53 to the nucleus to induce apoptosis (Chang et al., 2001, 2003, 2005; Lin et al., 2011; Tsai et al., 2013). Both p53 and WWOX work synergistically in inducing apoptosis. Without WWOX, p53 is destabilized and susceptible to ubiquitination and proteosomal degradation in the cytoplasm (Chang et al., 2005). When both WWOX and p53 are dysfunctional, osteosarcoma occurs in a double knockout mouse model, suggesting that both proteins participate in normal bone development (Del Mare et al., 2016). In another functional aspect, calcium ionophore/phorbol ester induces terminal maturation of MOLT-4 T cell acute lymphoblastic leukemia, which is due to Ser14 phosphorylation of WWOX, IκBα–ERK–WWOX signaling, and expression of CD3 and CD8 (Lin et al., 2011; Huang et al., 2016).

When WWOX undergoes Lys63-linked ubiquitination at Lys274 by E3 ubiquitin ligase ITCH, it relocates to the nucleus and enhances cell death (Abu-Odeh et al., 2014; Abu-Remaileh et al., 2015). In addition, vesicle trafficking protein TRAPPC6A binds WWOX and acts as a carrier for WWOX to undergo nuclear accumulation (Chang and Chang, 2015). Interestingly, WWOX can be subjected to E3 ligase polycomb2 (Pc2)-mediated SUMOylation, and this enables its suppression of DU145 prostate cancer tumorigenesis (Choi et al., 2015). Activated tyrosine kinase Cdc42-associated kinase (ACK1) promotes prostate cancer progression, via binding and phosphorylating WWOX at Tyr287 for polyubiquitination and proteosomal degradation (Mahajan et al., 2005).

Transiently overexpressed WWOX has been shown to block translocation of many transcription factors to the nucleus. And, this leads to a general belief that WWOX sequesters prosurvival transcription factors in the cytoplasm to block cancer growth (Chang et al., 2001, 2003, 2005, 2007; Aqeilan et al., 2004; Aqeilan and Croce, 2007; Aldaz et al., 2014; Abu-Remaileh et al., 2015). Inducible WWOX sequesters SMAD3 in the cytoplasm and inhibits its transcriptional function (Ferguson et al., 2013). Nonetheless, this does not work effectively *in vivo*. For example, endogenous WWOX rapidly binds and co-translocates with CREB to the nuclei of injured neurons during sciatic nerve transection in rats (Li et al., 2009). Endogenous WWOX does not block the nuclear translocation of CREB, NF-κB, c-Jun, and many other transcription factors (Li et al., 2009; Chang et al., 2014).

## HYAL-2 is a cognate receptor for TGF-β: signaling via HYAL-2-WWOX-SMAD4 to regulate cell growth or death

Epithelial cancer cells undergo epithelial-mesenchymal transition (EMT) to turn into malignancy. TGF-β-mediated EMT requires participation of HAS2 (Porsch et al., 2013). Overproduction of HA is needed to drive EMT via Twist and TGF-β–Snail signaling (Chanmee et al., 2014, 2016). TGF-β1 is growth-inhibitory to normal epithelial cells, whereas hyaluronidase PH-20 counteracts the effect of TGF-β1 (Chang, 1997), Chang (1998, 2002). TGF-β1 protects murine L929 fibroblasts from the cytotoxic effect of tumor necrosis factor (TNF), whereas transiently expressed HYAL-1 and HYAL-2 block the TGF-β1 function (Chang, 1998). The functional antagonism is associated, in part, with hyaluronidase-mediated rapid activation of ERK in L929 cells and TGF-β1 blocks the activation (Chang, 2001). In the canonical pathway, TGF-β1 binds membrane type II TGF-β receptor (TβRII) as a cognate receptor and then recruits TβRI, followed by phosphorylating SMAD2 and 3 and recruiting SMAD4 to translocate to the nuclei for controlling gene transcription (Hsu et al., 2009; Hata and Chen, 2016; Heldin and Moustakas, 2016).

Another explanation for the functional antagonism between TGF-β1 and hyaluronidases is that membrane HYAL-2 serves as a cognate receptor for TGF-β1 (Hsu et al., 2009). In a non-canonical pathway, binding of TGF-β to HYAL-2 results in recruitment of tumor suppressors WWOX and SMAD4 and their relocation into the nucleus (Hsu et al., 2009; Chang et al., 2010). By immunoelectron microscopy, we have shown that TGF-β1 binds membrane HYAL-2, followed by internalization of the resulting TGF-β1/HYAL-2 complexes by endosomes and fusion with lysosomes (Hsu et al., 2009). Whether the complex is degraded in the lysosome is unknown. The TGF-β1/HYAL-2 complexes must have triggered a membrane/intracellular machinery to allow the complex internalization. The WWOX/HYAL-2 complexes can be found on the cell surface by immunoelectron microscopy (Hsu et al., 2009). TGF-β1 also induces the internalization of the WWOX/HYAL-2 complexes. When WWOX and HYAL-2 are transiently overexpressed, TGF-β1 dramatically enhances the activation of SMAD responsive element (8–9-fold increases), which subsequently leads to cell death (>95% of promoter-activated cells). This raises the possibility that HYAL-2 and WWOX are cofactors for SMAD4 to bind the SMAD responsive promoter DNA and control gene expression.

Binding of WWOX with HYAL-2 and SMAD4 has been determined by co-immunoprecipitation, yeast two-hybrid analysis, immunoelectron microscopy, and real time FRET microscopy (Hsu et al., 2009, 2016). WWOX acts as a bridge to interact with both proteins. The first WW domain with Tyr33 phosphorylation is able to bind both HYAL-2 and SMAD4. However, the region(s) in HYAL-2 and SMAD4 for WWOX binding is unknown. SMAD4 also binds to the *C*-terminal SDR domain of WWOX. There is a competitive binding interaction between SMAD4 and HYAL-2 to the WW domain of WWOX (Hsu et al., 2016). Presumably, the HYAL-2–WWOX–SMAD4 complex is most stabilized for the binding of SMAD4 to the N-terminal WW domain of WWOX and HYAL-2 to the C-terminal SDR domain.

During traumatic brain injury, dramatic accumulation of the HYAL-2–WWOX complex is found in the apoptotic nuclei of damaged neurons in the brains of rats, suggesting a novel role of the overly loaded HYAL-2–WWOX complex in causing neuronal death *in vivo* (Hsu et al., 2016). Presumably, pericellular high molecular weight HA enhances the binding of TGF-β1 with HYAL-2 without transmitting the signal. Upon HA degradation by HYAL-2, TGF-β signaling starts. It has been reported that HA blocks TGF-β signaling by inducing trafficking of TGF-β receptors to lipid raft-associated pools, which facilitates increased receptor turnover (Ito et al., 2004a,b).

Taken together, there are key links that allow HYAL-2 to disrupt the TGF-β1 signaling. First, HA affects the binding of TGF-β1 with HYAL-2. Second, functional deficiency or defect of the HYAL-2-WWOX-SMAD4 signaling complex may occur in cancer cells. For example, tumor suppressor WWOX is frequently missing in cancer cells (Bednarek et al., 2000; Chang et al., 2001, 2003, 2007; Iliopoulos et al., 2005; Aqeilan and Croce, 2007; Chiang et al., 2013; Gardenswartz and Aqeilan, 2014; Abu-Remaileh et al., 2015; Schrock and Huebner, 2015; Huang et al., 2016). Lastly, the CD44-HYAL-2 complex provides spatial hindrance for the binding of TGF-β1 with Hyal-2. CD44 is not involved in the HYAL-2-WWOX-SMAD4 signaling (Hsu et al., 2009).

## High molecular weight HA induces bubbling cell death via signaling with overexpressed HYAL-2–WWOX–SMAD4 complex

Cytosolic WWOX is anchored, in part, at the cell membrane by HYAL-2 or Ezrin onto the cell membrane (Jin et al., 2006; Hsu et al., 2009). HYAL-2 is inserted on the cell membrane via a GPI linkage and is exposed onto the cell surface. WWOX is exposed onto the cell surface via binding with HYAL-2, as determined by immunoelectron microscopy (Hsu et al., 2009). It appears that WWOX by itself may localize on the cell surface (Hsu et al., 2009). However, how this works remain to be established. Whether membrane localization of WWOX is via interaction with other cytoskeletal proteins [e.g., merlin/NF2 and/or AMOT (Angiomotin) Abu-Remaileh et al., 2015], is unknown and remains to be established.

The fairly high molecular mass of HA in naked mole rats allows them to resist cancer growth (Tian et al., 2013). How HA blocks cancer growth is largely unknown. One report showed that HA-binding motifs of USP17 deubiquitinating enzyme and SDS3 (suppressor of defective silencing 3) are responsible for HA-mediated growth suppression (Ramakrishna et al., 2012). In addition, overexpression of endogenous HA leads to enhanced homotypic E-cadherin mediated cell-cell adhesion, induction of cell cycle inhibitors and suppression of G0/1 to S-phase transition of the cell cycle, and blocking of cell proliferation, (Bharadwaj et al., 2011). HA oligomers of approximately 2.5 × 10^3^ Daltons inhibit tumor growth *in vivo* (Ghatak et al., 2002). Also, these oligomers suppress anchorage-independent growth and apoptosis of cancer cells by blocking PI 3-kinase/Akt (protein kinase B) cell survival pathway and inducing caspase activation and apoptosis (Ghatak et al., 2002).

**Figure 1:**
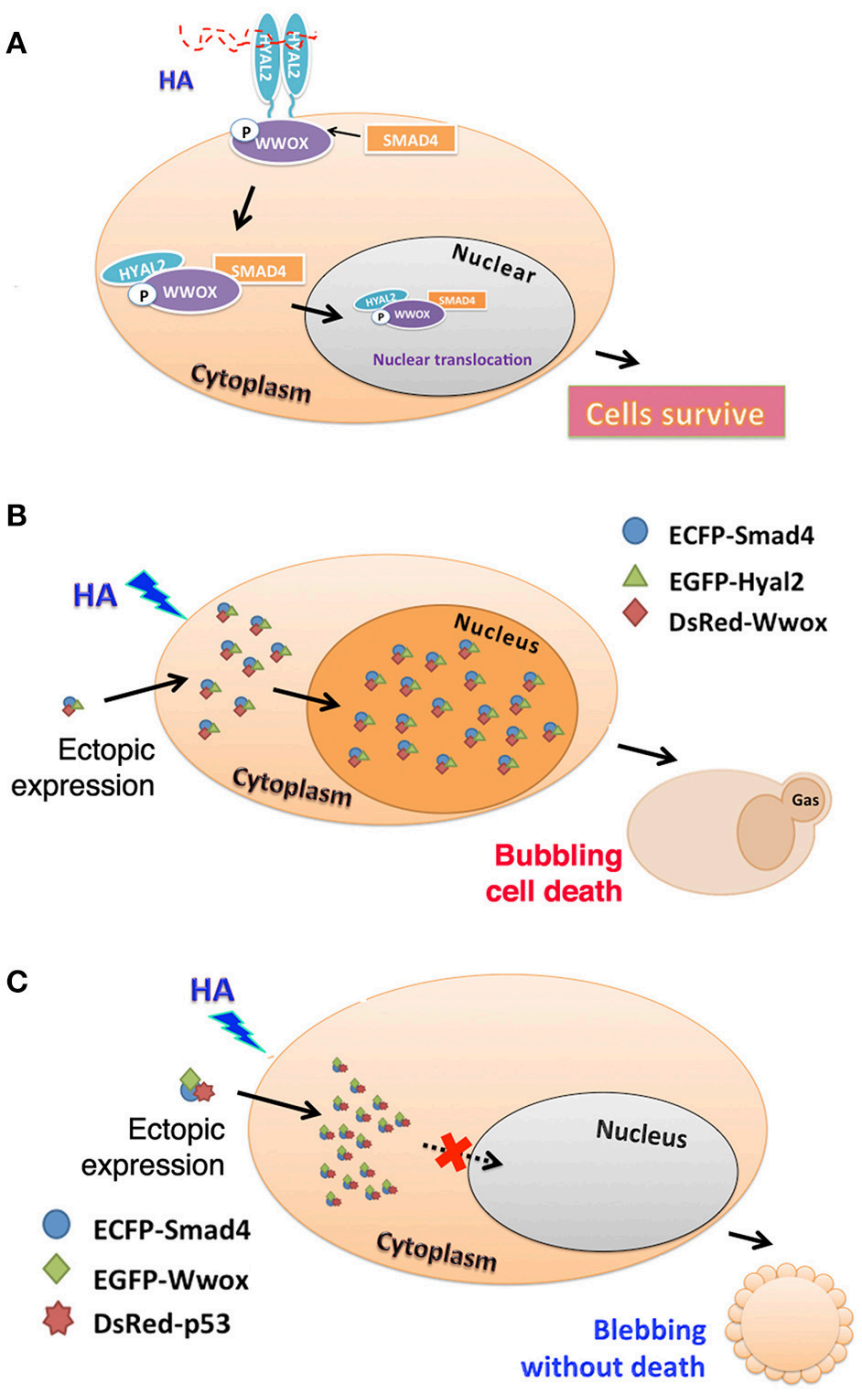
**HA activates endogenous HYAL-2–WWOX–SMAD4 signal pathway. (A)** A schematic graph for high molecular weight HA-induced signaling of HYAL-2–WWOX–SMAD4 complex is shown. Formation of the endogenous complex is increased with time. Relocation of the complex to the nucleus occurs in 20–40 min post stimulation. **(B)** As determined by time-lapse tri-molecular FRET microscopy (Hsu et al., 2016; Huang et al., 2016), HA activates the endogenous HYAL-2–WWOX–SMAD4 signaling and increases the complex formation of ectopic ECFP-SMAD4–EGFP-HYAL-2–DsRed-WWOX to undergo nuclear accumulation. Cells undergo bubbling cell death (Chen et al., 2015; Chang, 2016). **(C)** Also, HA induces the complex formation of ectopic ECFP-SMAD4—EGFP-WWOX—DsRed-p53, and membrane blebbing occurs in cells without leading to death. The complex fails to relocate to the nucleus.

We validated the HA-induced endogenous HYAL-2–WWOX–SMAD4 signaling by co-immunoprecipitation and time-lapse tri-molecular Förster resonance energy transfer (FRET) microscopy (Hsu et al., 2016). High molecular weight HA (2–3 million Daltons) induces a rapid increase in the formation of endogenous HYAL-2–WWOX–SMAD4 complex in cells (Figure 1A). Twenty to Fourty minutes later, the complex relocates to the nuclei in WWOX-expressing normal and cancer cells. In WWOX-deficient cells, SMAD4 spontaneously relocates to the nucleus without HA stimulation (Hsu et al., 2016). Stimulation of cells with HYAL-2 antibody, or HYAL-2 antisense expression cDNA, results in spontaneous accumulation of WWOX and SMADs in the nucleus (Hsu et al., 2016). Together, these observations suggest that endogenous HYAL-2 and WWOX regulate each other in a reciprocal manner and act together in blocking nuclear translocation of SMAD proteins.

HA-mediated signaling of endogenous HYAL-2–WWOX–SMAD4 drives the formation of ectopic complexes of ecfp-SMAD4–egfp-HYAL-2–DsRed-WWOX and ecfp-SMAD4–egfp-WWOX–DsRed-p53 (Hsu et al., 2016). HA increases the complex formation of ectopic ecfp-SMAD4–egfp-HYAL-2–DsRed-WWOX with time, and the complex relocates to the nucleus. Interestingly, cells undergo bubbling cell death (Figure 1B). Bubbling cell death is not identical to apoptosis, and has been defined as “formation of a single nitric oxide-containing bubble from the nucleus per cell and release of this swelling bubble from the cell surface to extracellular space that causes cell death.” (Chen et al., 2015; Chang, 2016). Bubbling cell death is caspase-independent, and exhibits no DNA fragmentation and flip-over of phosphatidylserine. Also, HA increases the complex formation of ectopic ecfp-SMAD4–egfp-WWOX–DsRed-p53with time, and the cytosolic complex localizes in the cytoplasm and causes membrane blebbing (Figure 1C). No cell death occurs. Failure of cell death is probably due to localization of the SMAD4–WWOX–p53 signaling complex in the cytoplasm. Malignant cancer cells are generally devoid of tumor suppressors WWOX, SMAD4, and p53. Or, they possess protein mutants. Conceivably, HA-induced signaling of HYAL-2–WWOX–SMAD4 is defective. This allows cancer cells gain growth advantage and degraded HA-increased metastasis. Calcium ionophore/phorbol ester induces the signaling of IκBα–ERK–WWOX for maturation of T cell leukemia, whereas HA has no effect in inducing the signaling event (Hsu et al., 2016; Huang et al., 2016).

## Zfra binds membrane HYAL-2 as a receptor in spleen HYAL-2^+^ CD3^−^ CD19^−^ Z lymphocyte

The HYAL-2–WWOX–SMAD4 signaling is involved in anticancer response *in vivo*. Zfra peptide binds membrane HYAL-2 as a receptor to activate a novel non-T/non-B spleen HYAL-2^+^ CD3^−^ CD19^−^ Z lymphocyte for memory anticancer response (Lee et al., 2015). Zfra, zinc finger-like protein that regulates apoptosis, is a naturally occurring 31-amino-acid protein (Hsu et al., 2005, 2008; Hong et al., 2007a; Degterev and Yuan, 2008; Dudekula et al., 2010; Lee et al., 2015). Like many peptide drugs, Zfra has a good potential in anticancer therapeutics (Li and Cho, 2010; Fosgerau and Hoffmann, 2015). Transiently overexpressed Zfra nullifies the functions of nuclear factor NF-κB, cJun *N*-terminal kinase 1 (JNK1), and tumor suppressors p53 and WWOX (Hsu et al., 2005; Hong et al., 2007a). Zfra participates in the pathway of mitochondrial apoptosis (Hsu et al., 2008). Synthetic Zfra peptide (e.g., Zfra1-31 or Zfra4-10) undergoes self-polymerization *in vivo* and *in vitro* (Lee et al., 2015). Polymerized Zfra peptide stays on the cell surface only. It cannot be internalized by cells and fails to induce cell death *in vitro* (Lee et al., 2015).

Zfra effectively prevents and blocks the growth of at least 10 types of cancer cells *in vivo* (Lee et al., 2015; Chang et al., 2016a,b). For example, T cell-deficient nude mice received an aliquot of full-length Zfra1-31 or Zfra4-10 at 2 mM in sterile PBS (or PBS only) via tail vein injections once per week for 3 consecutive weeks. After resting for 1 week, mice were then inoculated with skin basal cell carcinoma (BCC) cells (or other types of cancer cells) onto two subcutaneous sites in both flanks. Compared to controls, Zfra significantly blocked BCC growth by 50–75% (Lee et al., 2015). Alteration of a conserved phosphorylation site Ser8 to Gly8 abolishes the peptide polymerization and its anticancer effect *in vivo*, suggesting that Ser8 participates in peptide polymerization (Lee et al., 2015). Heterozygous *Wwox*^+/−^ mice with exon 2–4 ablation spontaneously develop tumor approximately at age one (Aqeilan et al., 2007), and Zfra4-10 completely cures the tumor in 50 days (Lee et al., 2015). Zfra is very powerful in blocking cancer metastasis and cancer stem cell development (Lee et al., 2015).

## Polymerized Zfra activates Z cell for memory anticancer response *in vivo*

**Figure 2:**
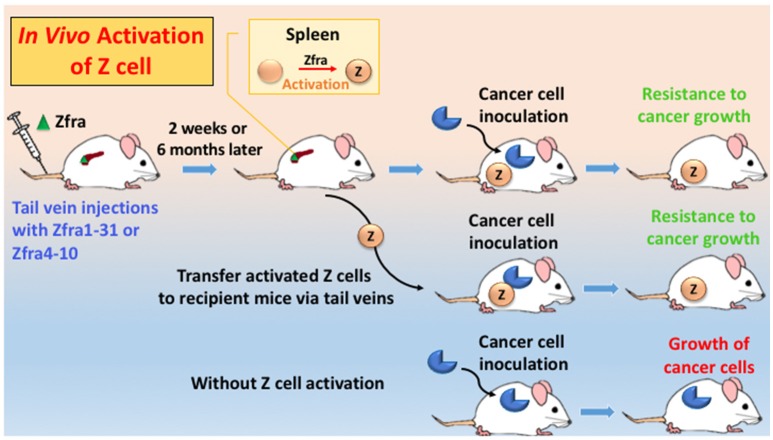
**An *in vivo* model for Zfra-mediated Z cell activation for cancer prevention and treatment**. Immune competent or deficient mice receive tail vein injections with full-length Zfra1-31 or Zfra4-10 peptide. Injected Zfra directly goes to the spleen but not other organs (Lee et al., 2015). Zfra activates spleen HYAL-2^+^ CD3^−^ CD19^−^ Z cell. Z cell probably goes to the cancer lesions to block tumor growth. Transfer of the activated Z cell to naïve mice or tumor-growing mice results in suppression of tumor growth. Without Z cell activation, tumor cells continue to grow.

Zfra effectively suppresses tumor-induced spleen splenomegaly by near 100% reduction (Lee et al., 2015). When mice receive Zfra peptides, either full length or Zfra4-10, via tail veins, the peptide is mainly trapped in the spleens but not in other organs (Lee et al., 2015). Zfra is not toxic to organs, including spleen, liver, lung, brain, skin, and other organs (Lee et al., 2015). Zfra-treated normal mice survive for the entire life span. Zfra activates memory spleen cells for cancer targeting via interacting with membrane HYAL-2 (Lee et al., 2015). When spleen cells are isolated from Zfra-treated nude mice, followed by transferring to naïve nude mice via tail vein injections, these cells confer resistance to cancer growth in the recipient mice (Figure 2; Lee et al., 2015). Naïve Z spleen cells are around 25–29% in the spleen of normal BALB/c mice, and spleen T and B cells 16 and 26%, respectively. In stark contrast, when tumor cells are growing in immune competent or deficient mice, these mice have 0.0–3.3% Z cells in the spleen (Lee et al., 2015). Binding of Zfra with HYAL-2 has been determined by co-immunoprecipitation and confocal microscopy (Lee et al., 2015). Importantly, Z cell can be activated *in vitro* by Zfra and works *in vivo* to prevent and block cancer growth (Lee et al., 2015).

## How does Z cell work *in vivo*?

“Immunization” of immune-competent and -deficient mice with Zfra peptides drives Z cell activation. No adjuvant is needed. Activated Z cells recognize many types of cancer cells, suggesting that there is a common antigen in the polymerized Zfra, which shares structural similarity with antigens on the surface of cancer cells. Notably, the Z cell level in the spleen drops dramatically down to 0–2% in tumor-growing mice. Whether Z cell has relocated to the cancer lesions is not known. Naïve Z cell is not “educated” or activated even in the presence of cancer antigens in the circulation. Accordingly, when spleen cells are isolated from naïve mice and then educated them with Zfra *in vitro*, the activated Z cell suppresses the ongoing growth of cancer in mice (Lee et al., 2015). Despite the so-called immunodeficient status, nude and NOD-SCID mice can be treated with Zfra to activate their Z cell populations to fight against cancer.

Both high molecular weight HA and TGF-β1 trigger the HYAL-2–WWOX–SMAD4 signaling (Hsu et al., 2009, 2016). Zfra induces capping of Hyal-2 on the cell surface (Hsu et al., 2009), and this may lead to WWOX phosphorylation at Tyr33 (p-WWOX) for interacting with HYAL-2, followed by internalization of HYAL-2–WWOX, along with Smad4. Perhaps, Zfra-mediated capping of Hyal-2 on the cell surface appears to be necessary to establish the induced memory anticancer response. Indeed, anti-Hyal-2 antibody and sonicated HA are able to achieve similar anticancer effects (Chang and Su, 2016). Sonicated HA has an altered that allows its strong binding with membrane HYAL-2 for provoking memory anticancer response.

## Conclusion and perspectives

Here, we detail the functional properties of HA, hyaluronidases and WWOX in normal physiology, pathological conditions, and cancer progression. We have also described TGF-β1 and HA-activated HYAL-2–WWOX–SMAD4 signaling, and the signaling related activation of SMAD responsive element and cell death. Whether the signaling is responsible for Zfra-mediated Z cell activation in conferring anticancer response *in vivo* is discussed. A likely scenario for Zfra-mediated Z cell activation is that Zfra binds membrane HYAL-2, which leads to recruit WWOX and then SMAD4. The resulting complex HYAL-2–WWOX–SMAD4 translocates to the nucleus for gene transcription. Alternatively, Zfra binds directly to membrane-localized WWOX (Hsu et al., 2009). Supporting evidence from yeast two-hybrid and FRET analyses revealed that non-activated WWOX is a “closed” form due to the binding of *N*-terminal WW domains with *C*-terminal SDR domain. Upon activation, Tyr33 becomes phosphorylated that leads to formation of an “open” form with WW and SDR domains in a dissociated state. Presumably, binding of Zfra to WWOX leads to Tyr33 phosphorylation and Ser14 de-phosphorylation, and the open form has an exposure of WWOX7-21 motif for Z cell activation. We have established that Tyr33-phosphorylated WWOX is an activated form for cancer suppression (Chang et al., 2001, 2003, 2007; Chiang et al., 2013; Abu-Remaileh et al., 2015; Huang et al., 2016). Nevertheless, how the extracellular polymerized Zfra peptide regulates the specific site phosphorylation in WWOX remains to be established.

## Author contributions

WS: Contributed one quarter in writing manuscript; IL: Managed references cited; YY, HK: Carried out graphic arts; CS, YY, LH, and MC: Proof read the manuscript and discussions; LH: Carried out graphic arts; NC: Wrote and proof read the manuscript, and performed graphic arts.

### Conflict of interest statement

The authors declare that the research was conducted in the absence of any commercial or financial relationships that could be construed as a potential conflict of interest.
